# Reclamation of Marine Chitinous Materials for Chitosanase Production via Microbial Conversion by *Paenibacillus macerans*

**DOI:** 10.3390/md16110429

**Published:** 2018-11-02

**Authors:** Chien Thang Doan, Thi Ngoc Tran, Van Bon Nguyen, Anh Dzung Nguyen, San-Lang Wang

**Affiliations:** 1 Department of Chemistry, Tamkang University, New Taipei City 25137, Taiwan; doanthng@gmail.com (C.T.D.); tranngoctnu@gmail.com (T.N.T.); 2 Department of Science and Technology, Tay Nguyen University, Buon Ma Thuot 630000, Vietnam; bondhtn@gmail.com; 3 Institute of Biotechnology and Environment, Tay Nguyen University, Buon Ma Thuot 630000, Vietnam; nadzungtaynguyenuni@yahoo.com.vn; 4 Life Science Development Center, Tamkang University, New Taipei City 25137, Taiwan

**Keywords:** chitin, chitosan, protease, chitinase, chitosan oligomers

## Abstract

Chitinous materials from marine byproducts elicit great interest among biotechnologists for their potential biomedical or agricultural applications. In this study, four kinds of marine chitinous materials (squid pens, shrimp heads, demineralized shrimp shells, and demineralized crab shells) were used to screen the best source for producing chitosanase by *Paenibacillus macerans* TKU029. Among them, the chitosanase activity was found to be highest in the culture using the medium containing squid pens as the sole carbon/nitrogen (C/N) source. A chitosanase which showed molecular weights at 63 kDa was isolated from *P. macerans* cultured on a squid pens medium. The purified TKU029 chitosanase exhibited optimum activity at 60 °C and pH 7, and was stable at temperatures under 50 °C and pH 3-8. An analysis by MALDI-TOF MS revealed that the chitosan oligosaccharides (COS) obtained from the hydrolysis of water-soluble chitosan by TKU029 crude enzyme showed various degrees of polymerization (DP), varying from 3–6. The obtained COS enhanced the growth of four lactic acid bacteria strains but exhibited no effect on the growth of *E. coli*. By specialized growth enhancing effects, the COS produced from hydrolyzing water soluble chitosan with TKU029 chitinolytic enzymes could have potential for use in medicine or nutraceuticals.

## 1. Introduction

Chitosan is a polysaccharide consisting of 1,4-ß-linked d-glucosamine residues, partially substituted with *N*-acetyl group. Recently, chitosan oligomer conversion has attracted attention among many researchers, because chitosan oligosaccharides are not only water-soluble, but also show various functional properties such as anti-inflammatory [1], anti-oxidative [2], anti-tumor [1,3], preservative [4], and prebiotic [5,6]. Chitosanase is a useful and environmentally-friendly tool for depolymerizing chitosan into oligosaccharides with various degrees of polymerization (DP). The major sources of chitosanase are bacteria, such as *Bacillus*, *Serratia*, *Aeromonas*, *Streptomyces*, *Pseudomonas*, and *Paenibacillus*. Almost all of these chitinolytic-producing bacteria were reported as using chitin or chitosan as the source of carbon/nitrogen (C/N). Commercialized chitin and chitosan products are mostly prepared from shrimp shells, crabs shells, or squid pens by chemical treatment to remove the mineral salts and proteins from these fishery processing by-products. In order to save on enzyme production cost, and in reutilizing the residual proteins, these chitin and protein-containing raw materials have been reported as the sole C/N source for enzyme production by *Bacillus* [7,8,9,10], *Pseudomonas* [11,12,13], and *Serratia* [14,15].

Until now, exopolysaccharides [16,17,18], α-glucosidase inhibitors [19,20], antioxidants [21], and biosurfactants [16] produced by *Paenibacillus* sp. growing on squid pens, shrimp shells, or crab shells have been reported; however, there have been few reports about chitinase production of *Paenibacillus* species on these low-cost chitinous materials. In the previous report [22], demineralized crab shells or squid pens have been used as the sole C/N source for comparing the activities of chitinase, chitosanases, proteases, and α-glucosidase inhibitors by 16 chitinolytic/proteolytic enzymes-producing strains, i.e., *Paenibacillus* sp. TKU029, *Paenibacillus* sp. TKU032, *Paenibacillus* sp. TKU037, *Paenibacillus* sp. TKU042 [22], *Bacillus licheniformis* [10], *Bacillus cereus* [2], *Serratia marcescens* [15], *Serratia ureilytica* [14], *Pseudomonas aeruginosa* [13]. Among these tested strains, *Paenibacillus* sp. TKU029 together with *Paenibacillus* sp. TKU042 produced the highest chitosanase activity when squid pens were used as the sole C/N source.

In this study, four kinds of the marine byproducts, i.e., squid pen powder (SPP), demineralized crab shell powder (deCSP), demineralized shrimp shell powder (deSSP), and shrimp head powder (SHP), were used as the sole C/N source to explore the production of chitosanase by *P. macerans* TKU029 via fermentation. The purification and characterization of these TKU029 chitosanase were performed. The oligomers obtained by hydrolyzing water-soluble chitosan with TKU029 chitosanase were analyzed by MALDI-TOF-MS, and their enhancing effect on the growth of lactic acid bacteria was also estimated.

## 2. Results and Discussion

### 2.1. Screening of Chitinous Materials as Sole C/N Source for Chitosanase Production

**Figure 1:**
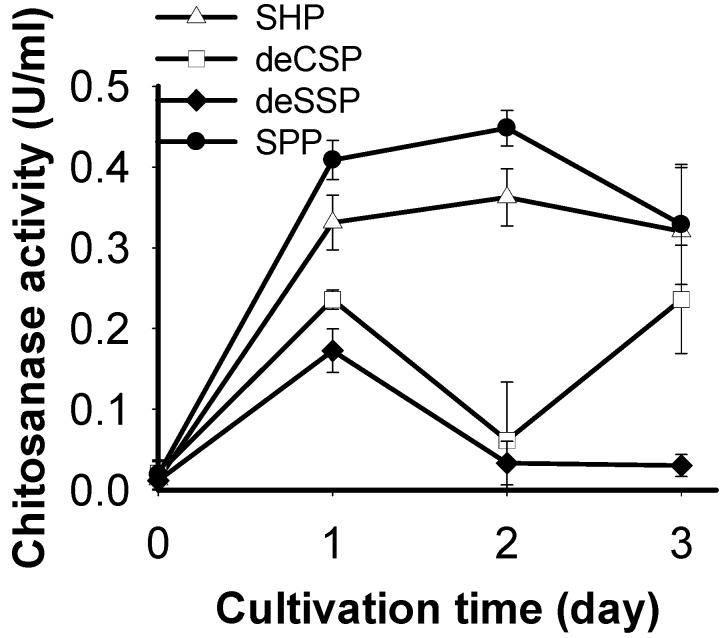
Production of chitosanase by *P. macerans* TKU029 using different chitin-containing materials as the C/N source. SHP, shrimp head powder; deCSP, demineralized crab shell powder; deSSP, demineralized shrimp shell powder; SPP, squid pen powder. The error bars represent standard deviations (*n* = 3).

Chitinous materials have been proposed as an important factor for producing chitinase/chitosanase by fermentation [22]. Therefore, four containing-chitin marine byproducts (SHP, SPP, deCSP, and deSSP) were investigated for the production of chitinase by *P. macerans* TKU029 in this study. As shown in Figure 1, the highest chitosanase productivity by *P. macerans* TKU029 was observed with the C/N source of SPP (0.448 ± 0.022 U/mL, 2 day). These results were consistent with the research of Doan et al. [22], which showed that *Paenibacillus* sp. TKU042 produced the highest chitosanase activity on SPP (day 2).

SPP, with its high ratio of protein and low ratio of mineral salts, was claimed to be a good C/N source for producing exopolysaccharides and a bio surfactant by *P. macerans* TKU029 [16]. In this study, SPP was also found to be the best C/N source for chitinase production by *P. macerans* TKU029. 

### 2.2. Purification and Characterization of Chitosanase

**Figure 2:**
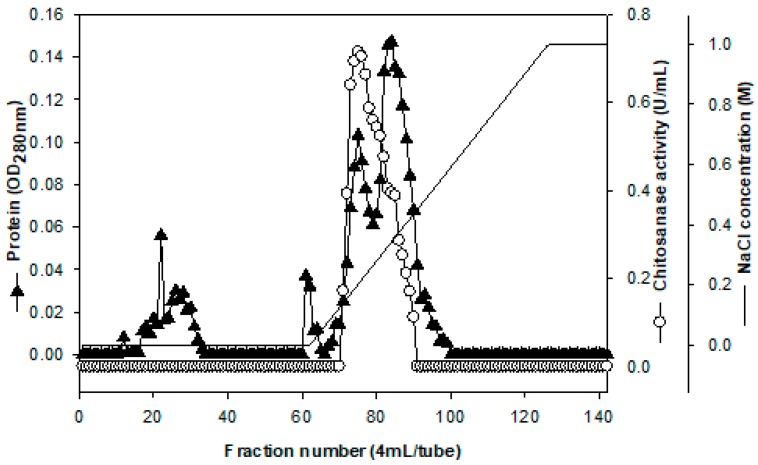
A typical elution profile of chitosanase on DEAE-Sepharose CL-6B column. The column was equilibrated with 50 mM phosphate buffer (pH 7) at a flow rate of 3 mL/min and 4 mL/fraction.

In order to explore the enzyme characterization and make a comparison with other reports, the chitosanase was purified from the culture supernatant (600 mL) of *P. macerans* TKU029 by ethanol precipitation and ion exchange chromatography of DEAE-Sepharose CL-6B. As shown in Figure 2, one chitosanase was eluted with a linear gradient of 0-1 M NaCl in the same buffer. The eluted fractions of chitosanasewas pooled for further purification by chromatography of Macro-Prep DEAE, respectively. Table 1 summarize the purification results of TKU029 chitosanase, respectively. TKU 029 chitosanase was purified from the culture supernatant with the weight recovery 1.43 mg, respectively. The final specific activity and recovery yields of TKU029 chitosanase were 24.19 U/mg and 10.51%, respectively (Table 1).

**Figure 3:**
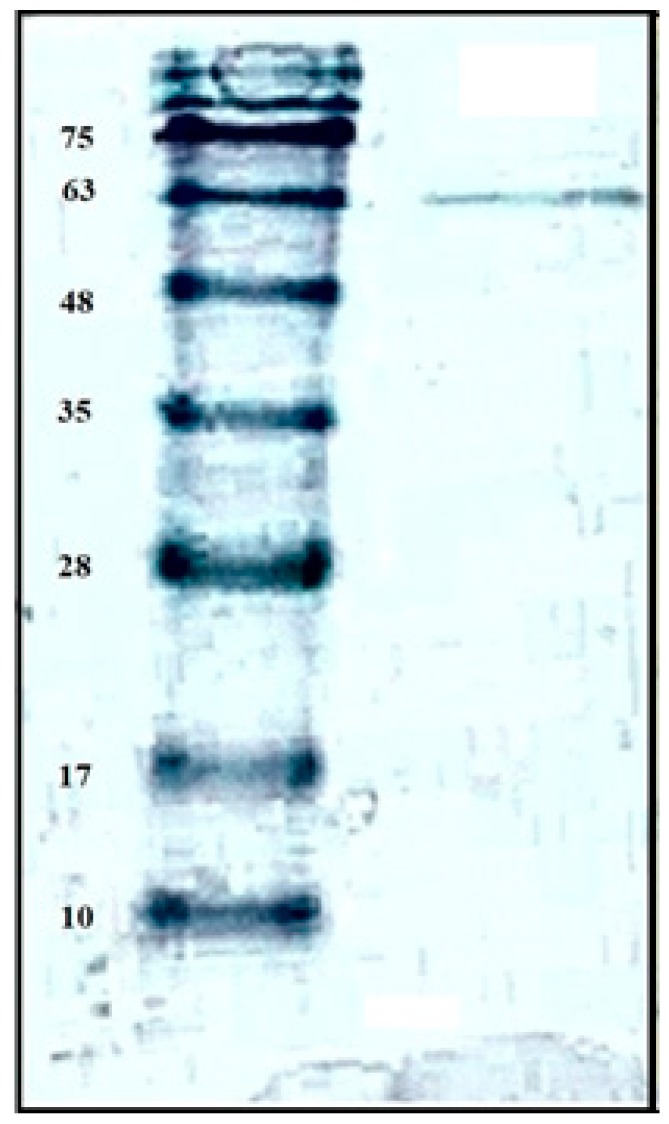
SDS-PAGE analysis of the chitosanase produced by TKU029.

Similar to most of the other chitinolytic enzymes-producing strains of *Paenibacillus* species, only one chitinase or chitosanase were purified from the culture supernatant [22,23,24,25,26,27,28,29,30,31,32,33]. However, the molecular mass of TKU029 chitosanase (63 kDa) estimated by SDS-PAGE (Figure 3) differed from the chitinases or chitosanases of the other *Paenibacillus* strains, for instance, *P. pasadenensis* NCIM5434 chitinase (35 kDa) [23], *Paenibacillus* sp. 1794 chitosanase (40 kDa) [24], *P. thermoaerophilus* TC22-2b chitinase (48 kDa) [25], *P. larvae* ATCC9545 (49 kDa) [26], *P. illinoisensis* KJA-424 chitinase (54 kDa) [33], *Paenibacillus* sp. D1 chitinase (56 kDa) [27], *P. barengoltzii* CAU904 chitinase (67 kDa) [28], *P. elgii* HOA73 chitinase (68 kDa) [29], *P. pasadenensis* CS0611 chitinase (69 kDa) [30], *Paenibacillus* sp. TKU042 chitosanase (70 kDa) [22], *Paenibacillus* sp. D2 chitosanase (85 kDa) [31], and *Paenibacillus* sp. FPU-7 (150 kDa) [32].

Chitinolytic enzyme productions from *Paenibacillus* strains on a colloidal-containing chitin medium have been widely reported [23,25,27,33], but rarely on a containing-SPP medium [22]. By using squid pen, a marine byproduct, as the C/N source for microbial cultivation, the production cost of microbial chitosanase could be reduced. In this study, a chitosanase from *P. macerans* TKU029 were isolated by SPP conversion. To the best of our knowledge, this is the first report about chitosanase production from *P. macerans* species using the medium containing SPP.

### 2.3. Effects of pH and Temperature on Activity and Stability of Chitosanase

**Figure 4:**
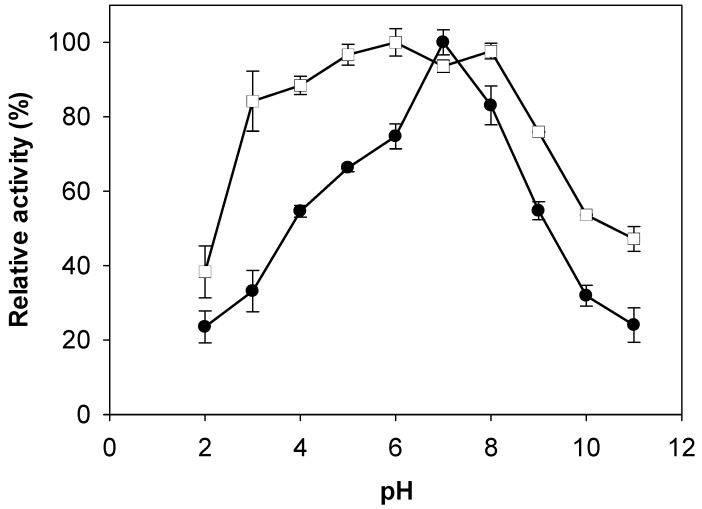
Effect of pH on activity and stability of TKU029 chitosanase. (●), enzyme activity; (□), enzyme stability. The error bars represent standard deviations (*n* = 3).

The effect of pH on the activities of TKU029 chitosanase was studied herein (Figure 4). The optimum pH for TKU029 chitosanase was pH 7. Compared to chitinase/chitosanase from other *Paenibacillus* strains, the optimum pH of TKU029 chitinase differed from most reports, which showed enzyme optimum activity on acid condition; for instance, *Paenibacillus* sp. D1 [27], *P. pasadenensis* CS0611 [30], and *P. illinoisensis* KJA-424 [33] were pH 5; *Paenibacillus* sp. 1794 was pH 4.8 [24]; *P. barengoltzii* was pH 3.5 [28]; *P. thermoaerophilus* TC22-2b was pH 4 [25]; and *Paenibacillus* sp. M4 was pH 6.5, but it was consistent with *P. elgii* HOA73 (pH 7) [29]. These results also observed TKU029 chitosanase to have broad pH stability (pH 3–8). Several *Paenibacillus* strains chitinases/chitosanases have broad pH stability close to that of TKU029 [28,29,34].

**Figure 5:**
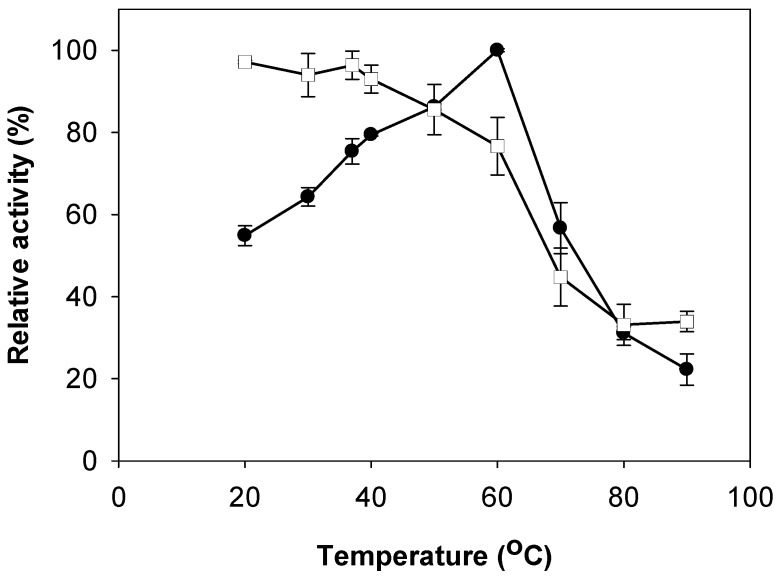
Effect of temperature on activity and stability of TKU029 chitosanase. (●), enzyme activity; (□), enzyme stability. The error bars represent standard deviations (*n* = 3).

The optimum temperature and thermal stability of TKU 029 chitosanasewere also investigated (Figure 5). The optimum temperature of TKU029 chitosanase was 60 °C; it remained stable up to 50 °C. At the optimum temperature of 60 °C, the chitosanase still retained 76% activity. However, the activity was dramatically decreased to less than half of the highest activity when the temperature was above 60 °C. Generally, the optimum temperature of TKU029 chitosanase is higher than those of other *Paenibacillus* strains, such as *P. pasadenensis* NCIM 5434 [23], *Paenibacillus* sp. D1 [27], *P. elgii* HOA73 [29], *P. pasadenensis* CS0611 [30]. Due to the higher optimum temperature, it is suggested that TKU029 chitosanase may be suitable for industrial application.

### 2.4. Effect of Metal Ions on Activity of Chitosanase

To further explore the effect of some ion metals on their activities, TKU029 chitosanase were prepared in 50 mM phosphate buffer (pH 7) containing 5 mM of each chemical and incubated at 37 °C in 10 min. The mixtures were then examined for their residual activities. The results are summarized in Table 2. TKU029 chitosanase activity was not affected by Zn^2+^ and Ca^2+^, but other chemicals had clear effects on the enzyme. In the presence of Cu^2+^, Mg^2+^, Ba^2+^ and EDTA, the activity of TKU029 chitinase was dramatically reduced with 63.42%, 64.31%, 76.99%, and 68.10% residue activity. Interestingly, these results also showed that the addition of 5 mM Na^+^ and Fe^2+^ into the enzyme solution could enhance chitinase activity (154.22% and 133.23%). Similarly, Meena et al. [34], also observed the increased activity with Fe^2+^ and Na^+^ in *Paenibacillus* sp. BRSR-047 chitinase.

### 2.5. Substrate Specificity of Chitosanase

The substrate specificity of TKU029 chitosanase was also investigated. As shown in Table 3, TKU029 chitosanase exhibited the most activity on water soluble chitosan, followed by chitosan, colloidal chitin and β-chitin, but with no activity on the α-chitin and non-chitin substrates. These results indicated that the rate of hydrolysis was strongly affected by the physical form of the substrate. In addition, since the enzyme expressed no activity on *p*-nitrophenyl-*N*-acetyl-*β*-d-glucosaminide (*p*NPG), a substrate used for analyzing exochitinase, TKU029 chitosanase could be initially classified as a endochitosanase.

### 2.6. Chitosan Hydrolysis and COS Production

**Figure 6:**
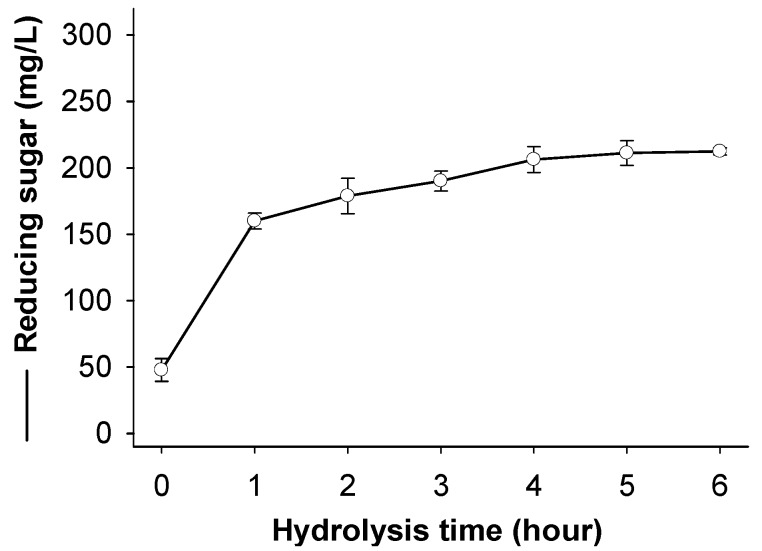
Hydrolysis time course measurement of reducing sugar with TKU029 chitosanase. The reaction mixture in a 250 mL Erlenmeyer flask containing 1% water soluble chitosan in 100 mL phosphate buffer (pH 7, 50 mM) and 10 mL of crude enzyme. The hydrolysis condition was carried at 50 °C in 6 h. The hydrolysis solution was then tested reducing sugar under the assay mentioned in the methods section. The error bars represent standard deviations (*n* = 3).

Since water soluble chitosan showed the most effect on TKU029 chitosanase activity, the hydrolysis products from this substrate were also studied. The course of chitosan sample degradation was conveniently studied by the measurement reducing sugar. Figure 6 shows the reducing sugar of the sample as a function of reaction time. The reducing sugar dramatically increased in the early stage of the reaction (after 1 h of reaction) and did not increase after 4 h.

COS with low DP have been reported to exhibit several interesting bioactivities, for instance, antioxidant [2], antitumor [1,3], and prebiotic [5,6]. Thus, many studies have recently attracted interest for converting chitosan into chitooligosaccharides. Based on the obtained results, the chitosan hydrolysis by TKU029 crude enzyme was observed to possess great potential to produce chitosan oligosaccharide with low DP.

**Figure 7:**
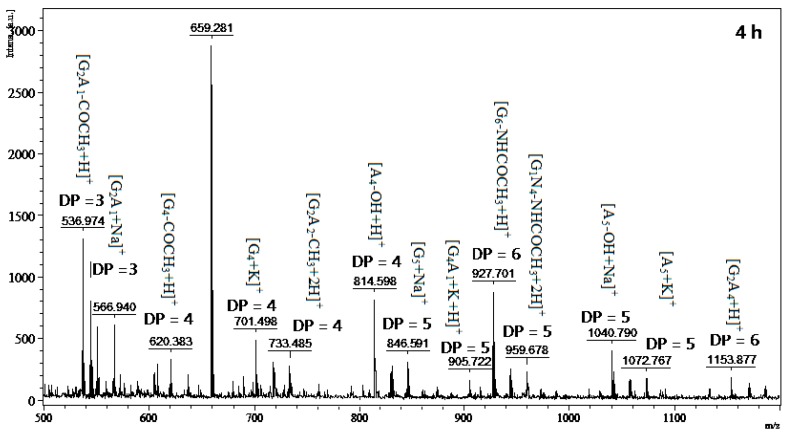
MALDI-TOF-MS of the oligomer mixtures obtained during water soluble chitosan hydrolysis. The proportion of low DP oligomer (DP < 7) was reduced by precipitation in the 90%methanol-soluble/90% acetone-insoluble fraction. The identified peaks are labeled with DP, in which DP indicates the degree of polymerization.

To obtain the low DP oligomers in the chitosan hydrolysis, selective precipitation in 90% methanol and acetone solutions was performed following the aforementioned method [2]. Since MALDI-TOF-MS analysis is limited to molecular masses higher than 500 Da, the DP < 2 oligomer could not be determined by this method. The product from the reaction with TKU029 crude enzyme is a mixture containing both homo-chitooligosaccharides, including (GlcN)_4_ (Short form: G_4_) (*m/z* 620, 701), G_5_ (*m*/*z* 846), G_6_ (*m*/*z* 927, 1040), (GlcNAc)_4_ (Short form: A_4_) (*m*/*z* 814), A_5_ (*m*/*z* 1072) and herero-chitooligosacharides, including G_2_A_1_ (*m*/*z* 536, 566), G_2_A_2_ (*m*/*z* 733), G_4_A_1_ (*m*/*z* 905, 959), G_2_A_4_ (*m*/*z* 1153) (Figure 7). Compared to other reports, COS with different broad DP had been produced by various microoganism strains, for instance, *B. cereus* TKU027 (DP 4–9, 2–5) [35], *B. cereus* TKU031 (DP 3–8) [9], *Penicillium janthinellum* D4 (DP 3–9) [36], *Aspergillus fumigatus* S-26 (DP 2–7) [33], and *Bacillus* sp. KFB-C108 (DP 3–5) [37], while this study observed the DP up to 6 and it was only similar to *B. cereus* TKU022 (DP 2–6) [38]. On the other hand, differing chitosan hydrolysates from other *Bacillus* strains [9,35,38], the originally-classified genus of *Paenibacillus*, the hydrolysis products by TKU029 also contained homo-chitosaccharides with higher ratio than hetero-chitosaccharides (at the same DP) (Figure 7). These results indicated that TKU029 chitosanase may hydrolyze the water soluble chitosan by cleavage of glycosidic bonds of the type –G|A– and–A|G–, whereas the hyrolysis of –G|G– and –A|A– are slow. From these results, a quick and simple method to obtain chitosan oligosaccharide with low DP (3–6) could be performed by combining the TKU029 chitosanase hydrolysis reaction with water soluble chitosan at substrate and a selective methanol/acetone precipitation.

### 2.7. Evaluation of Growth Enhancing Effect of COS on Lactic Acid Bacteria

**Figure 8:**
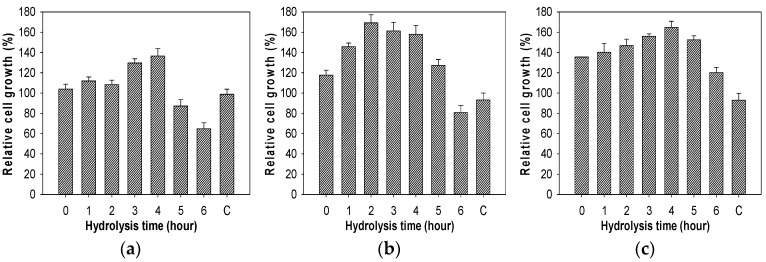

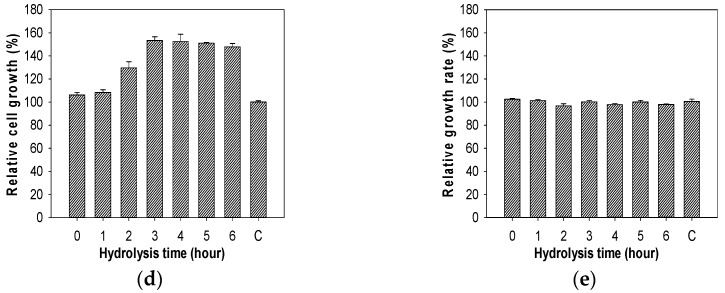
Effect of the chitosan hydrolysis on the growth of bacteria. (**a**), *L. lactis* BCRC 10791; (**b**), *L. paracasei* BCRC 14023; (**c**), *L. rhamnosus* BCRC 16000; (**d**), *L. rhamnosus* BCRC 10791; (**e**), *E. coli* BCRC 51,950. The error bars represent standard deviations (*n* = 3).

The effect of the chitosan oligosaccharides obtained from chitosan hydrolysis generated by TKU029 chitosanase on the growth of lactic acid bacteria were also studied. As shown in Figure 8, chitosan oligosaccharides, which were collected from different hydrolysis times, have a clear effect on the growth of lactic acid bacteria. The highest cell growth of *L. lactis* BCRC 10791 was found for the addition of 4-h hydrolyzed chitosan (136.59%), *L. paracasei* BCRC 14023 was 2 h (169.37%), *L. rhamnosus* BCRC 16,000 was 4 h (164.81%) and *L. rhamnosus* BCRC 10791 was 3 h (153.34%). Interestingly, adding 0.1% chitosan oligosaccharides into the medium did not increase the growth of *E. coli* BCRC 51950. These results differed to COS from other reports, which only showed the enhancing effect on lactic acid bacteria [5,6]. Due to a prebiotic requiring a selectivity effect on the growth of a limited group of bacteria, it is suggested that COS produced from TKU029 may have the potential to become a prebiotic candidate.

## 3. Materials and Methods

### 3.1. Materials

Squid pens, crab shells, and shrimp shells were collected from Shin-Ma Frozen Food Co. (I-Lan, Taiwan). Shrimp head powder (SHP) was gifted from Fwu-Sow Industry (Taichun, Taiwan). Demineralized shrimp shell powder (deSSP) and demineralized crab shell powder (deCSP) were prepared via acid treatment [22]. Chitin (from shrimp shell), tyrosine, D-glucosamine and the reagents (3,5-dinitrosalicylic acid and Folin-Ciocalteu) were all purchased from Sigma-Aldrich Corp. (Singapore). Macro-prep DEAE and Macro-Prep High S were bought from Bio-Rad (Hercules, CA, USA). All other reagents were the highest grade available. *P. macerans* TKU029 [16] was provided by Life Science Development Center, Tamkang University, Taiwan.

### 3.2. Measurement of Chitosanase Activity

The measurement of chitinase activity was performed according to a previously-described method [22], with modifications. Chitosan (1% in 50 mM phosphate buffer) was used as the substrate. The reaction was performed with 0.1 mL substrate and 0.1 mL enzyme solution, and kept at 37 °C for 30 min. The amount of reducing sugar produced in the supernatant was determined by DNS reagent, with D-glucosamine as the reference compound. One unit of enzyme activity was defined as the amount of enzyme that produced 1 µmol of reducing sugar per min.

### 3.3. Screening of Chitinous Materials as Sole C/N Source for Enzyme Activity

Four kinds of chitinous materials, i.e., crab shell powder (deCSP), squid pen powder (SPP), shrimp head powder (SHP), and demineralized shrimp shell powder (deSSP), were used as the sole sources of C/N (1%, *w*/*v*). *Paenibacillus macerans* TKU029 was grown in 100 mL of liquid medium in 250 mL Erlenmeyer flasks containing 1% of each chitinous material, 0.1% K_2_HPO_4_ and 0.05% MgSO_4_·7H_2_O. The incubation was performed with 1% of seed culture, in 3 d at 37°C in a shaking incubator (150 rpm). During each of 24 h, the culture medium was collected for further measurements.

### 3.4. Purification of Chitosanae

*P. macerans* TKU029 was cultured in 100 mL of liquid medium in an Erlenmeyer flask (250 mL) containing 1% SPP, 0.1% K_2_HPO_4_ and 0.05% MgSO_4_.7H_2_O in a shaking incubator for 3 days at 37 °C to collect culture supernatant. A protein precipitation step was performed by adding 1800 mL of cold ethanol (−20 °C) to 600 mL of culture supernatant and kept at 4 °C overnight. To collect the crude enzyme, the precipitate was centrifuged at 12,000× *g* for 30 min and then dissolved in a small amount of 50 mM phosphate buffer (pH 7). The obtained crude enzyme was loaded onto a DEAE-Sepharose CL-6B column that had been equilibrated with 50 mM phosphate buffer. The obtained chitosanase (eluted by a linear gradient of 0–1 M NaCl in the same buffer) fractions were then further purified by A Macro-Prep DEAE column, respectively. The molecular mass of the purified enzymes was determined using the SDS-PAGE methods.

### 3.5. Effects of pH and Temperature on Activity and Stability of Chitosanase

The optimal temperature for the enzymatic reaction was performed at different points of temperature (20–100 °C) during 30 min. To assess the thermal stability, the enzyme solution was treated at a range of temperature during 15 min. The treated enzyme was then used to measure the residual activity.

The optimal pH of enzyme was measured in buffers of different pH values (pH 2–11). To determine the effect of pH on enzyme stability, the enzyme was incubated in buffer of different pH levels at 37 °C for 30 min, and the residual activity was measured at optimal pH value. The buffer systems used included glycine.HCl (50 mM, pH 2–3), acetate (50 mM, pH 4–5), phosphate (50 mM, pH 6–8) and Na_2_CO_3_-NaHCO_3_ (50 mM, pH 9–11).

### 3.6. Effect of Metal Ions on Chitosanase Activity

Several metal ion salts (5 mM in final concentration) were used to investigate their effect on enzyme activity, i.e., CuCl_2_, ZnCl_2_, MgCl_2_, NaCl, BaCl_2_, CaCl_2_, FeCl_2_, and EDTA.

### 3.7. Substrate Specificity

Chitosanase was incubated in 50 mM phosphate buffer with various kinds of substrates at 60 °C for 30 min. α-chitin (from shrimp shell), β-chitin (from squid pen), chitosan (DD = 65.36% and MW = 350,000–500,000 Da), water soluble chitosan (DD = 66.66% and MW = 30,000 Da), colloidal chitin (from α-chitin), cellulose, dextran (70,000 Da), Starch (from potato) were included. The chitinase activity in colloidal chitin was used as a control to calculate the relative activity of enzyme in other substrates.

### 3.8. MALDI-TOF MS Analysis

A sample (1 μL) was prepared with 1 μL of 2,5-dihydroxybenzoic acid as a matrix in H_2_O-CAN-TFA solution (50/50/0.1%, *v*/*v*/*v*). Positive ion mode of MALDI mass spectra was acquired with a MALDI-TOF instrument (Bruker Daltonics, Bremen, Germany) equipped with a nitrogen laser emitting at 337 nm operating in linear mode. Each mass spectrum was the accumulated data of approximately 30–50 laser shots. External 3-point calibration was used for mass assignment.

### 3.9. Growth Enhancing Effect of COS on Lactic Acid Bacteria Test

Four lactic acid strains were chosen for the experiment: *Lactobacillus lactis* BCRC 10791, *Lactobacillus paracasei* BCRC 14023, *Lactobacillus rhamnosus* BCRC 16,000, and *Lactobacillus rhamnosus* BCRC 10,791. The bacteria were cultured in MRS medium containing 0.1% (*w*/*v*) chitosan oligosaccharides for 24 h at 37 °C. To examine whether chitosan oligosaccharides could affect non-lactic acid bacteria, a strain of *E. coli* BCRC 51,950 was also cultured in LB medium containing 0.1% (*w*/*v*) chitosan oligosaccharides under the same condition with lactic acid bacteria. A measurement of optical density 600 nm of culture supernatant was used to calculate the cell growth of the bacteria.

## 4. Conclusions

Among the four marine chitinous materials, *Paenibacillus macerans* TKU029 achieved the best result for chitosanase production using squid pens as the sole carbon/nitrogen source. The molecular weight of the purified TKU029 chitosanase (63 kDa) was different from those of the other *Paenibacillus* strains. The oligomers obtained by hydrolyzing water soluble chitosan with TKU029 chitosanase possessed specifically enhancing effects on the growth of analyzed four lactic acid bacteria, and had no effect on *E. coli*. By the selective growth enhancing effect of COS, TKU029 chitosanase has potential to be used in the production of nutraceuticals.

## Figures and Tables

**Table 1:** Purification of the chitosanase from *P. macerans* TKU029.

Step	Total Protein (mg)	Total Activity (U)	Specific Activity (U/mg)	Recovery (%)	Purification (Fold)
**Culture supernatant**	1245.88	328.09	0.26	100.00	1.00
**EtOH precipitation**	60.31	83.90	1.39	25.57	5.28
**DEAE-Sepharose CL-6B**	10.45	63.17	6.04	19.25	22.95
**Macro-Prep DEAE**	1.43	34.48	24.19	10.51	91.87

**Table 2:** Effect of metal ions on the activity of chitosanase.

	Relative Activity (%)
Control	100.00 ± 1.39
Cu^2+^	63.42 ± 1.51
Zn^2+^	99.75 ± 1.84
Mg^2+^	64.31 ± 2.09
Na^+^	154.22 ± 1.96
Ba^2+^	76.99 ± 2.03
Ca^2+^	108.81 ± 4.13
Fe^2+^	133.23 ± 5.31
EDTA	68.10 ± 0.39

All data points were means ± standard deviations (*n* = 3).

**Table 3:** Substrate specificity of TKU029 chitosanase.

Substrate	Relative Activity (%)
Chitosan	100 ± 16.93
Water soluble chitosan	196.43 ± 15.55
α-Chitin	0
β-Chitin	12.30 ± 6.62
Colloidal Chitin	63.26 ± 4.08
*p*NPG	0
Cellulose	0
Dextran	0
Starch	0

All data points were means ± standard deviations (n = 3).
